# Association between dietary niacin intake and Helicobacter pylori seropositivity in US adults: A cross-sectional study

**DOI:** 10.1371/journal.pone.0308686

**Published:** 2024-08-15

**Authors:** Zeru Chen, Shixin Wu, Guangzhan Chen, Xuguang Guo

**Affiliations:** 1 Department of Clinical Laboratory Medicine, Guangdong Provincial Key Laboratory of Major Obstetric Diseases, Guangdong Provincial Clinical Research Center for Obstetrics and Gynecology, The Third Affiliated Hospital, Guangzhou Medical University, Guangzhou, China; 2 Department of Clinical Medicine, The Second School of Clinical Medicine, Guangzhou Medical University, Guangzhou, China; 3 Department of Preventative Medicine, School of Public Health, Guangzhou Medical University, Guangzhou, China; 4 Department of Clinical Medicine, The Sixth School of Clinical Medicine, Guangzhou Medical University, Guangzhou, China; 5 Department of Clinical Medicine, The Third School of Clinical Medicine, Guangzhou Medical University, Guangzhou, China; 6 Guangzhou Key Laboratory for Clinical Rapid Diagnosis and Early Warning of Infectious Diseases, King Med School of Laboratory Medicine, Guangzhou Medical University, Guangzhou, Guangdong, China; School of Pharmacy, Ardabil University of Medical Sciences, ISLAMIC REPUBLIC OF IRAN

## Abstract

**Objectives:**

This study delves into the association between dietary niacin intake and Helicobacter pylori seropositivity, a topic gaining prominence in academic discourse. However, the precise role of Niacin in the development and progression of Helicobacter pylori seropositivity remains inadequately understood. Thus, this research aims to investigate the connections between H. pylori seropositivity and dietary niacin intake using a nationally representative sample of adults.

**Methods:**

A cross-sectional analysis encompassed 4,000 participants from the National Health and Nutrition Examination Survey (NHANES) conducted in the United States between 1999 and 2000, all aged 20 years or older. The study employed the generalized additive model (GAM) and multivariate logistic regression to explore the potential relationship between niacin intake and Helicobacter pylori seropositivity. Subgroup analyses were performed based on gender, age, diabetes, hypertension, and hyperlipemia.

**Results:**

Analyzing cross-sectional data from NHANES 1999–2000 involving individuals aged 20 years and above revealed that out of 4,000 participants, 1,842 tested positive for H. pylori via serology. Multivariate analyses unveiled a significant inverse correlation between niacin intake and H. pylori seropositivity. Adjusted odds ratios (ORs) for dietary niacin intake in quartiles Q2 (13.31–19.26 mg/d), Q3 (19.27–27.42 mg/d), and Q4 (>27.42 mg/d) compared to Q1 (<13.31 mg/d) were 0.83 (95% CI: 0.69–1.01), 0.74 (95% CI: 0.61–0.90), and 0.66 (95% CI: 0.54–0.81), respectively. Moreover, a nonlinear L-shaped relationship (P = 0.022) emerged between niacin intake and H. pylori seropositivity, indicating minimal risk of H. pylori infection at approximately 44.69 mg of niacin per day in the diet.

**Conclusion:**

This study suggests a potential link between increased dietary niacin intake and reduced prevalence of Helicobacter pylori seropositivity. This correlation is bolstered by plausible mechanisms involving immunomodulatory function, mitochondrial dysfunction, and cellular oxidative stress.

## 1. Introduction

Helicobacter pylori, often referred to as H. pylori, is a spiral-shaped, gram-negative bacterium known for colonizing the human stomach’s mucosa [1]. It’s significantly linked to gastrointestinal conditions like gastritis, peptic ulcers, and gastric cancer [2]. Despite extensive research, it remains prevalent, affecting an estimated 4.4 billion people globally in 2015, imposing a substantial burden on public health systems, highlighting the urgency for preventive strategies [3, 4]. Recent studies exploring dietary influences on H. pylori infection suggest potential preventive dietary interventions, albeit with specific components requiring further investigation [5–7].

Niacin, also known as vitamin B3, is a water-soluble B vitamin found in various foods and supplements [8], derived from both plant and animal sources such as fortified cereals, meats, and vegetables [9]. Its role in NAD-related diseases has expanded its perceived function beyond being solely a vitamin, prompting exploration into potential therapeutic applications [10]. As a precursor of NAD and NADP [11], nicotinic acid serves as a crucial cofactor in mitochondrial energy metabolism [10]. Its deficiency can impede oxidative phosphorylation and disrupt mitochondrial respiration [12]. Clinical investigations suggest that increasing niacin intake through diet may enhance immunity and potentially reduce the colonization of H. pylori [13–16]. Previous studies have indicated that mitochondrial dysfunction, oxidative stress, and immune modulation may influence the pathogenesis and progression of H. pylori infection [17–19]. However, there is a lack of comprehensive and sizable cross-sectional research exploring the potential correlation between dietary niacin intake and the presence of H. pylori antibodies.

Therefore, this study aims to fill this gap by examining the relationship between dietary niacin consumption and H. pylori seropositivity using the extensive National Health and Nutrition Examination Survey (NHANES) database. Understanding this connection could unveil new paths for preventing H. pylori infection.

## 2. Methods

### 2.1 Date sources and study design

This study utilized a cross-sectional research approach, drawing on data from the NHANES, a comprehensive, multistage, stratified, and complex survey conducted nationally by the Centers for Disease Control and Prevention (CDC) [20]. NHANES provides demographic information, dietary assessments, health interviews, physical exams, and laboratory tests for the US ambulatory population [21, 22]. Participants’ dietary status was evaluated through two-day, 24-hour dietary recalls, while physical examinations and blood samples were collected at a mobile examination center (MEC). Detailed information about the survey and associated research data is available on the NHANES website at https://www.cdc.gov/nchs/nhanes/.

**Fig 1:**
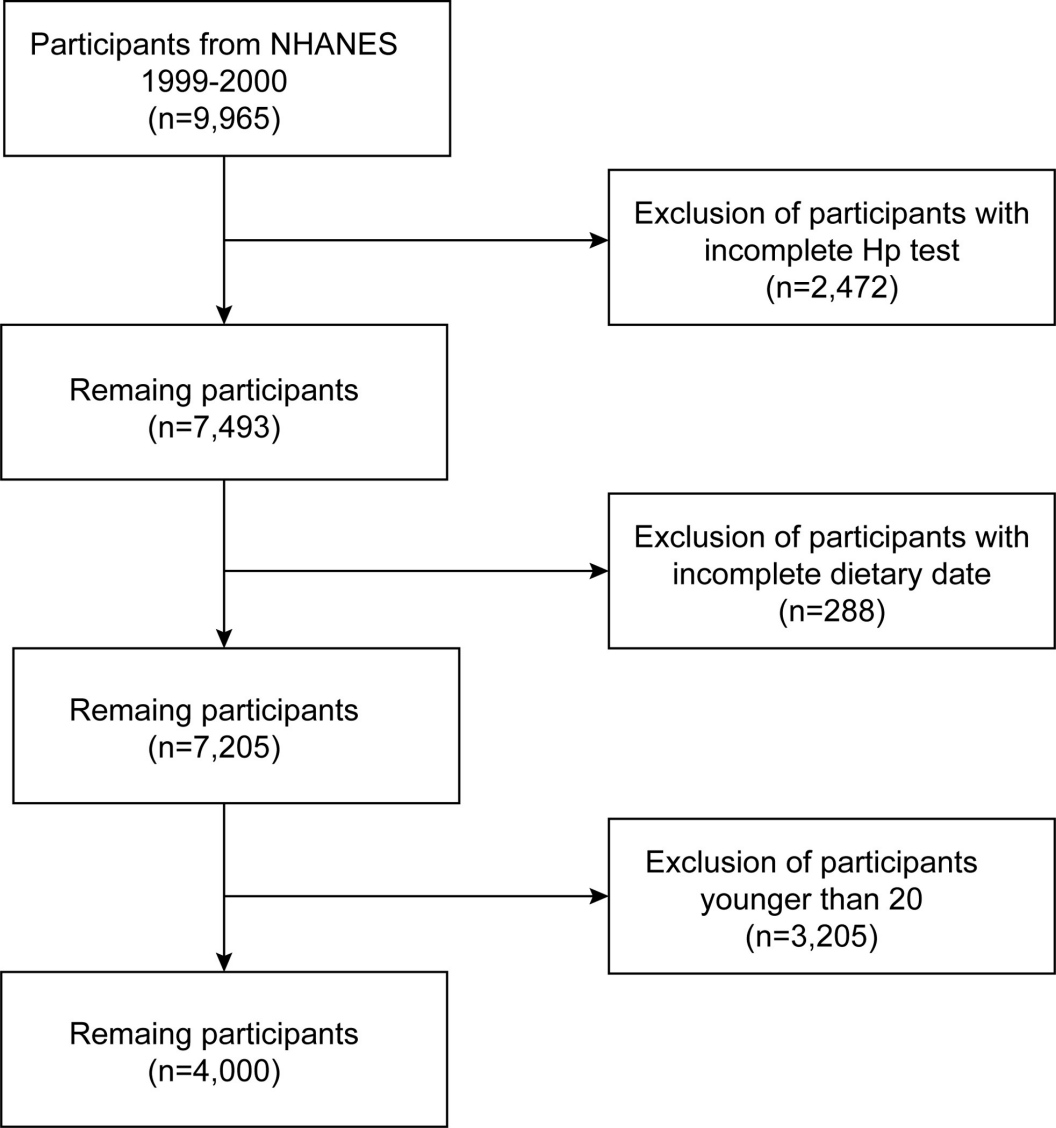
Flowchart detailing the selection process for patients included in this analysis.

The survey employed the 1999–2000 US NHANES dataset. Initially comprising 9,965 eligible participants, the study excluded 2,472 due to incomplete H.p data, 288 due to incomplete dietary niacin data, and 3,205 under the age of 20. Ultimately, 4,000 participants were included. The flowchart in **Fig 1** outlines the sample selection process. The 1999–2000 NHANES protocol was approved by the National Center for Health Statistics Research Ethics Review Board. Before participating in the study, all subjects provided informed written consent [23]. Our study rigorously adhered to the protocols outlined by the Strengthening the Reporting of Observational Studies in Epidemiology (STROBE) criteria [24].

### 2.2 Dietary niacin intake

The study on dietary niacin intake involved face-to-face interviews at the Mobile Examination Center (MEC) [25]. Eligible NHANES participants underwent two 24-hour dietary recall interviews, initially at the MEC conducted by trained personnel and followed up 3 to 10 days later via phone or mail [26, 27]. The dietary recall method used in NHANES is designed to capture detailed information on all foods and beverages consumed by the participants within the past 24 hours. This method ensures comprehensive dietary data collection, which is then processed and validated by NHANES staff.

Nutrient analysis utilized the U.S. Department of Agriculture’s Food and Nutritional Dietary Studies Database (FNDDS). The dataset summarized individual nutrient intake, with niacin intake calculated as the average of the two dietary reviews. Niacin intake, including supplementation, was assessed following NHANES guidelines. Categorization into quartiles (Q1–Q4) and continuous variables represented different levels of intake, with critical values at 25%, 50%, and 75% of the total sample niacin intake. Quartiles were delineated as Q1 (<13.31 mg/day), Q2 (13.31–19.26 mg/day), Q3 (19.27–27.42 mg/day), and Q4 (>27.42 mg/day).

### 2.3 Helicobacter pylori antibody measurement

Helicobacter pylori antibody levels were measured using an enzyme-linked immunosorbent assay (ELISA) for H. pylori IgG by Wampole Laboratory in Cranbury, NJ [28]. Immunological status ratios (ISR) were calculated by dividing the average cutoff value by the average optical density (OD) of the test sample [29]. Ratio Diagnostics in Frankfurt, Germany, developed the ELISA for qualitative detection and measurement of H. pylori IgG antibodies in human blood. The serum anti-H. pylori assay determined specific antibody titers, with levels above 0.9 indicating positive infection in this study [30].

### 2.4 Covariates

Covariates, chosen based on clinical expertise and previous literature [31–33], encompassed age, gender, education, body mass index (BMI), poverty-to-income ratio (PIR), marital status, smoking and alcohol status, diabetes, and cardiovascular disease indicators. Gender was categorized as male or female, education as below high school, high school, or above high school, and marital status as married/cohabiting or living alone [34]. Alcohol and smoking patterns were treated as binary variables, defined respectively as consuming at least 12 drinks per year and classifying never smokers as those who never smoked or smoked fewer than 100 cigarettes in their lifetime [35]. BMI categories were established as normal weight (BMI <25 kg/m^2^), overweight (BMI ≥25, <30 kg/m^2^), and obese (BMI ≥30 kg/m^2^) [36]. Diabetes was identified in participants with a past or current diagnosis [37], while cardiovascular disease was determined based on reported history [38]. Specific data on serum albumin, total cholesterol, creatinine, and C-reactive protein (CRP) were extracted from NHANES laboratory test results.

### 2.5 Statistical analysis

The R statistical package (version 3.6.3; R Foundation) and EmpowerStats program (version 4.1) were utilized for data analysis. Weighted percentages were applied to categorical variables, while descriptive analyses for continuous variables involved averages, standard deviations (SD), or median and quartile range (QR) to characterize the study population. Kruskal-Wallis and chi-square tests assessed statistical significance for continuous and categorical variables, respectively. The association between dietary niacin intake and Helicobacter pylori (H. pylori) infection was indicated by OR values.

Multifactorial logistic regression explored this association, creating a corrected model accounting for included covariates. Trends were identified through quartile median calculations. Model 1 adjusted for age, sex, marital status, education, and PIR. Model 2 was fully adjusted, encompassing blood cardiovascular disease, smoking, drinking, diabetes, body mass index, and serological indices (CRP, creatinine, total cholesterol, and albumin). Dietary niacin intake was analyzed as both continuous and categorical variables in multivariate logistic regression, categorizing niacin intake into Q1 (<13.31 mg/day), Q2 (13.31–19.26 mg/day), Q3 (19.27–27.42 mg/day), and Q4 (>27.42 mg/day), with Q1 as the reference group.

Addressing the non-linear correlation, a generalized additive model with smooth curve fitting was used, alongside a recursive algorithm and two-stage logistic model to determine the inflection point. Interaction analysis involved likelihood ratio tests and hierarchical logistic regression among age, gender, diabetes, hypertension, and hyperlipemia subgroups. The Wald statistic tested trend variable heterogeneity, while cross-product terms determined subgroup membership. A two-sided p-value <0.05 indicated statistical significance with 95% confidence intervals. The study adhered to the Strengthening the Reporting of Observational Studies in Epidemiology (STROBE) declaration for cross-sectional studies.

## 3. Result

### 3.1 Baseline characteristics population

The study utilized NHANES data from 1999 to 2000. After applying strict exclusion criteria (e.g., missing H. pylori data, unknown dietary niacin intake, age <20 years), a final cohort of 4,000 participants underwent analysis. **Fig 1** visually represents the study outcomes and specific exclusion criteria.

Univariate analyses revealed associations between H. pylori seropositivity and variables such as age, sex, education, poverty-to-income ratio (PIR), body mass index (BMI), alcohol use, diabetes mellitus, serum albumin, heart failure, cardiac assault, and niacin intake (**S1 Table**).

**Table 1** displays the descriptive characteristics of participants. Among the 1,842 individuals with H. pylori infection, the average age was 49.8 years, and 46.8% were male. Notably, this group differed from the H. pylori seronegative group in various aspects: lower niacin intake, higher age, lower education and income levels, increased BMI, elevated serum c-reactive protein levels, higher diabetes mellitus prevalence, and increased occurrences of hypertension, heart failure and heart attack. Furthermore, the H. pylori seropositive group had a higher proportion of non-alcohol users compared to the seronegative group.

**Table 1:** The baseline characteristics and demographic characteristics of the study population (N = 4000).

Variables	Total (n = 4000)	Helicobacter pylori Seronegativity (n = 2158)	Helicobacter pylori Seropositivity (n = 1842)	*P* value
Gender, n (%)				0.046
Male	1871 (46.8)	978 (45.3)	893 (48.5)	
Female	2129 (53.2)	1180 (54.7)	949 (51.5)	
Age, Mean ± SD	49.8 ± 18.7	47.2 ± 18.9	52.9 ± 18.1	< 0.001
Education, n (%)				< 0.001
Below High School	1543 (38.6)	501 (23.2)	1042 (56.6)	
High School	897 (22.4)	546 (25.3)	351 (19.1)	
Above high school	1560 (39.0)	1111 (51.5)	449 (24.4)	
Marital Status, n (%)				0.263
Living alone	1516 (37.9)	835 (38.7)	681 (37)	
Married or living with a partner	2484 (62.1)	1323 (61.3)	1161 (63)	
PIR, n (%)				< 0.001
<1.3	1072 (26.8)	434 (20.1)	638 (34.6)	
≥1.3	2928 (73.2)	1724 (79.9)	1204 (65.4)	
BMI, n (%)				0.005
<25	1276 (31.9)	734 (34)	542 (29.4)	
≥25,<30	1415 (35.4)	727 (33.7)	688 (37.4)	
≥30	1309 (32.7)	697 (32.3)	612 (33.2)	
Serum indicators				
Creatinine, Mean±SD	0.8 ± 0.6	0.8 ± 0.6	0.8 ± 0.6	0.972
Albumin, Mean±SD	4.4 ± 0.4	4.4 ± 0.4	4.4 ± 0.3	< 0.001
Total cholesterol, Mean±SD	199.5 ± 40.8	198.6 ± 40.1	200.6 ± 41.7	0.13
CRP,Mean±SD	0.5 ± 1.0	0.5 ± 1.0	0.6 ± 1.0	0.046
Smoke status, n (%)	1874 (46.9)	981 (45.5)	893 (48.5)	0.056
Alcohol, n (%)	2632 (65.8)	1470 (68.1)	1162 (63.1)	< 0.001
Diabetes, n (%)	381 (9.5)	152 (7)	229 (12.4)	< 0.001
Cardiovascular disease				
Hypertension, n (%)	1214 (30.3)	592 (27.4)	622 (33.8)	< 0.001
Hyperlipemia, n (%)	1363 (34.1)	708 (32.8)	655 (35.6)	0.067
Heartfailure, n (%)	121 (3.0)	46 (2.1)	75 (4.1)	< 0.001
Coronary disease, n (%)	158 (4.0)	75 (3.5)	83 (4.5)	0.095
Angina,n (%)	147 (3.7)	69 (3.2)	78 (4.2)	0.082
Heart attack, n (%)	174 (4.3)	78 (3.6)	96 (5.2)	0.014
Dietary intake				
Niacin,Mean ± SD	21.9 ± 12.9	23.3 ± 13.0	20.3 ± 12.5	< 0.001

%, weighted proportion;

Hp, Helicobacter pylori; CRP, C-reactive protein; BMI: Body Mass Index;

PIR: Poverty Income Ratio.

Cardiovascular disease (hypertension, hyperlipemia, heartfailure, coronary disease, angina, and heart attack).

Dietary intake (Niacin).

### 3.2 The connection between dietary niacin intake and H.p seropositivity

In examining the association between niacin intake as a continuous variable and H. pylori seropositivity, a notable inverse relationship was observed. Each 1 mg/d increase in niacin intake was significantly associated with a reduced chance of H. pylori seropositivity across all models—unadjusted, model 1, and model 2—yielding consistent odds ratios (OR) of 0.98 (95% CI 0.98–0.99), 0.99 (95% CI 0.98–0.99), and 0.99 (95% CI 0.98–0.99) respectively, indicating a robust correlation between higher niacin intake and decreased H. pylori seropositivity.

Furthermore, when niacin intake was categorized into quartiles and adjusted for various demographic and health-related variables, a substantial inverse association between dietary niacin consumption and H. pylori seropositivity was established. Compared to individuals with lower niacin consumption (Q1, <13.31 mg/day), those in Q2 (13.31–19.26 mg/day), Q3 (19.26–27.42 mg/day), and Q4 (>27.42 mg/day) displayed adjusted OR values of 0.83 (95% CI: 0.69–1.00, p = 0.0600), 0.74 (95% CI: 0.61–0.90, p = 0.0028), and 0.66 (95% CI: 0.54–0.81, p < 0.0001) respectively (**Table 2**). Notably, trend tests confirmed the statistical significance of the overall decreasing trend in H. pylori seropositivity with increasing dietary niacin intake (p<0.0001).

**Table 2:** Multivariable logistic regression to assess the association of niacin intake with Helicobacter pylori seropositivity.

	Unadjusted model		Model I		Model II	
	OR (95% CI)	*P* value	OR (95% CI)	P value	OR (95% CI)	P value
niacin	0.98(0.98~0.99)	<0.0001	0.99(0.98~0.99)	0.0002	0.99(0.98~0.99)	0.0003
niacin quartile						
Q1	1.0 [Ref]		1.0 [Ref]		1.0 [Ref]	
Q2	0.75 (0.63~0.89)	0.0013	0.84 (0.69~1.01)	0.0699	0.83 (0.69~1.01)	0.0600
Q3	0.60 (0.50~0.72)	<0.0001	0.74 (0.61~0.90)	0.0021	0.74 (0.61~0.90)	0.0028
Q4	0.48 (0.40~0.57)	<0.0001	0.66 (0.54~0.81)	<0.0001	0.66 (0.54~0.81)	<0.0001
*P* for trend	0.97 (0.97~0.98)	<0.0001	0.98 (0.98~0.99)	<0.0001	0.87 (0.82~0.93)	<0.0001

Niacin enter as a continuous variable per 1 mg/day increase

Unadjusted model: no covariates were adjusted

Model I: Adjusted for age,gender,marital status,poverty-income ratio and education.

Mode II: Adjusted for age,gender,marital status,poverty-income ratio,education,body mass index,cardiovascular disease (hypertension,hyperlipemia,heartfailure,coronary heart disease,angina and heart attack),smoking,alcohol, diabetes, and serum indicators (CRP,albumin,total cholesterol and creatinine).

CI, confidence interval; OR, odds ratios; Ref, reference.

Niacin is in the quartile

Q1(<13.31mg/day),Q2(13.31–19.26mg/day),Q3(19.27–27.42mg/day),Q4(>27.42mg/day)

### 3.3 Dose-response relationships

**Fig 2:**
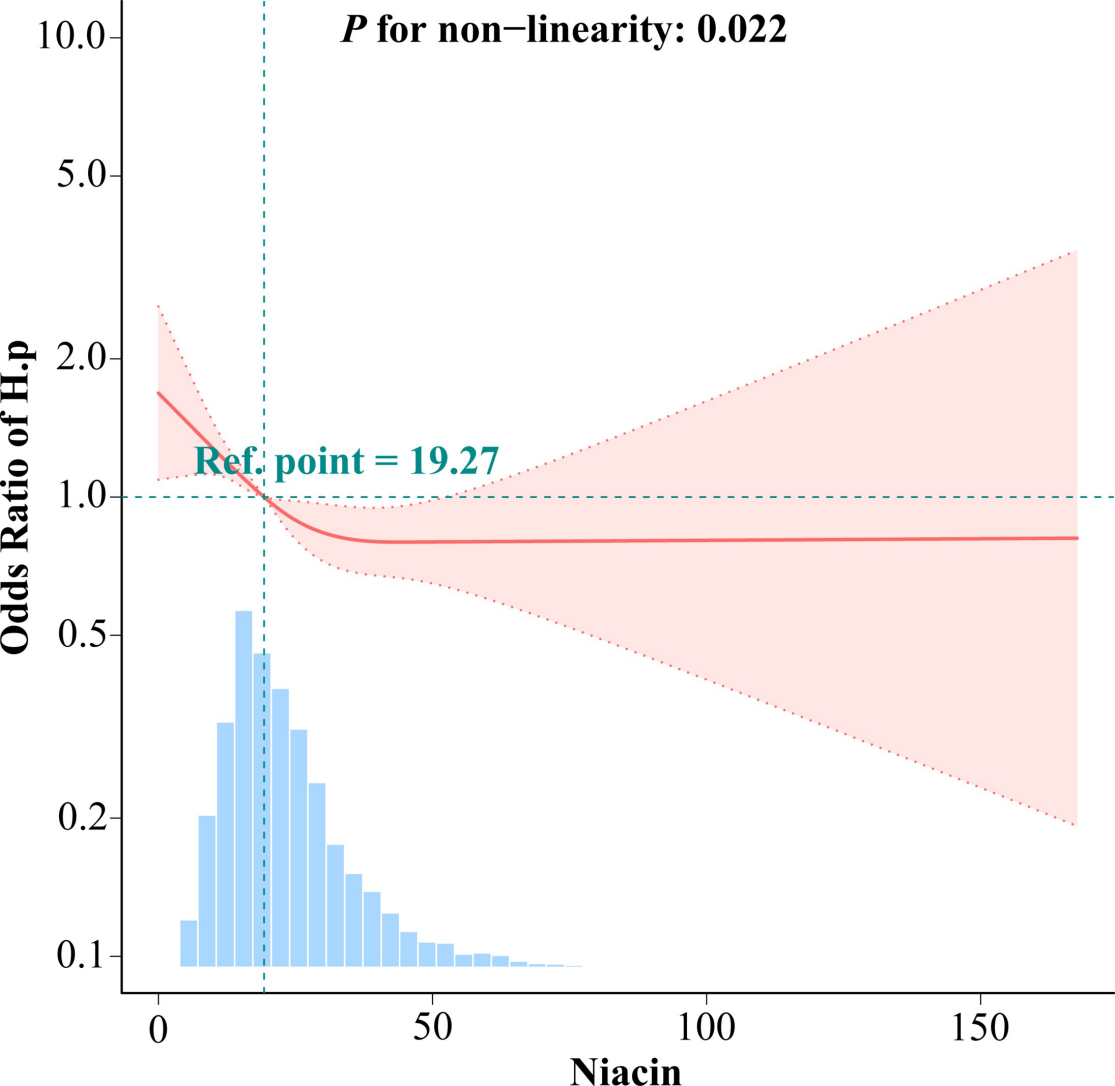
Dose–response relationship between dietary niacin intake and Helicobacter pylori seropositivity odds ratio. Solid and dashed lines represent the predicted value and 95% confidence intervals. Adjusted for age, gender, marital status, poverty-income ratio, education, body mass index, cardiovascular disease (hypertension, hyperlipemia, heartfailure, coronary heart disease, angina, and heart attack), smoking, alcohol, diabetes, and serum indicators (CRP, albumin, total cholesterol, and creatinine). Only 99% of the data is shown. H.p, Helicobacter pylori; CI, confidence interval; OR, odds ratios; Ref, reference.

The study investigated dietary niacin intake as a continuous variable. A significant non-linear relationship (p = 0.022) resembling an "L" shape was observed between dietary niacin intake and H. pylori seropositivity, as revealed by multivariate-adjusted restricted triple spline analysis (**Fig 2**). Through graphical analysis and clinical relevance, the optimal inflection point for niacin intake was determined to be 44.69 mg/day. Among individuals consuming less than 44.69 mg of niacin daily, a 1 mg/day increase in niacin corresponded to a 2.0% reduction in H. pylori infection risk (CI: 0.98 [0.97–0.99]; p < 0.0001). **Table 3** illustrates that there was no significant association for niacin intakes equal to or greater than 44.69 mg/day (odds ratio [OR], CI: 1.01 [1.00–1.03]; p = 0.1476).

**Table 3:** Threshold effect analysis of relationship of niacin intake and Helicobacter pylori seropositivity.

	Niacin	
	Adjusted OR(95%CI)	*P-*value
Two model		
Niacin intake < 44.69	0.98(0.97,0.99)	<0.0001
Niacin intake ≥ 44.69	1.01(1.00,1.03)	0.1476
Non-linear test		0.022

Adjusted for age, gender, marital status, poverty-income ratio, education, body mass index,cardiovascular disease (hypertension,hyperlipemia,heartfailure, coronary heart disease, angina, and heart attack), smoking, alcohol, diabetes, and serum indicators(CRP,albumin, total cholesterol, and creatinine).Hp, Helicobacter pylori; CRP, C-reactive protein; CI, confidence interval; OR, odds ratios;Ref, reference.

### 3.4 Subgroup analysis

**Fig 3:**
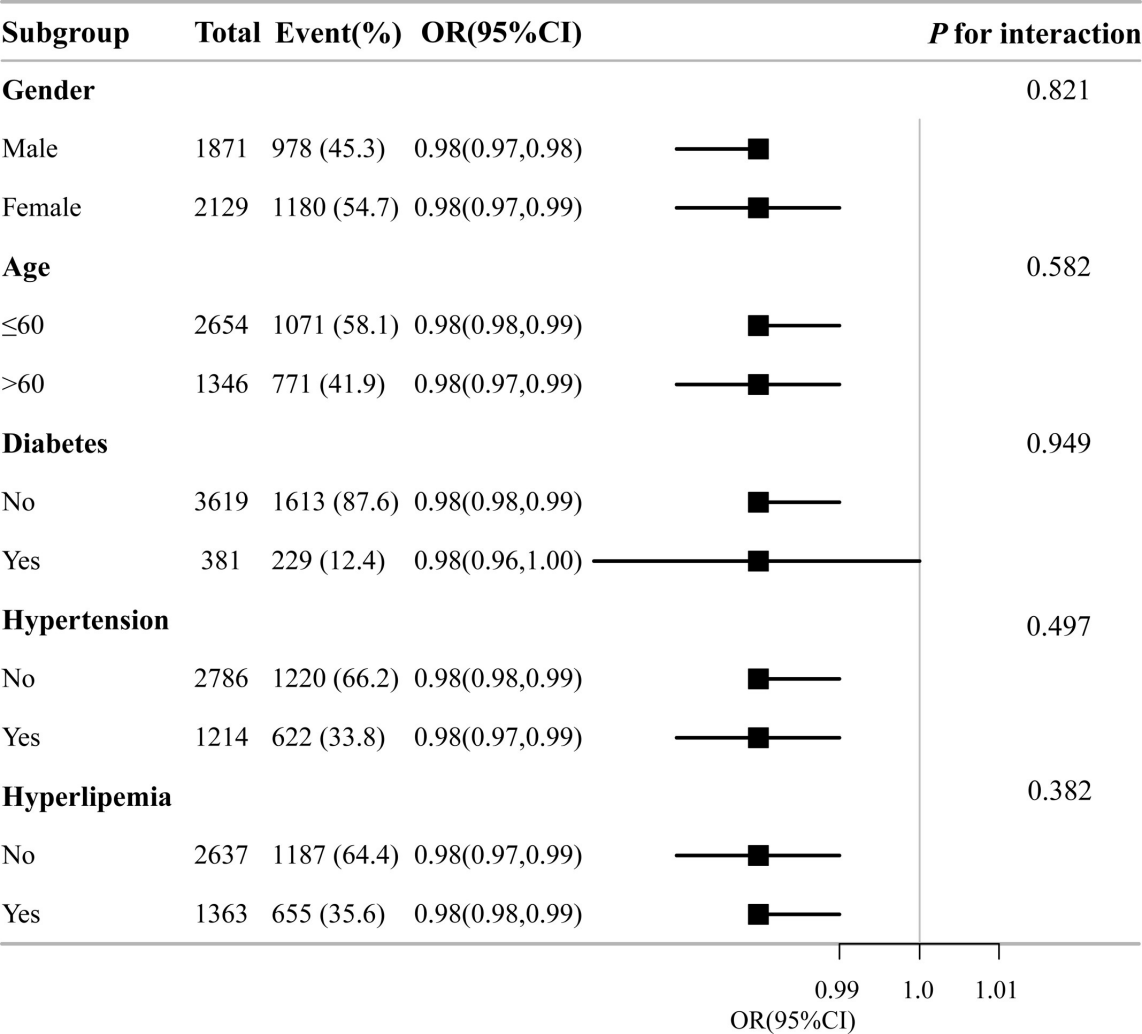
Subgroup analysis association between dietary niacin intake and Helicobacter pylori seropositivity. Adjusted for age, gender, marital status, poverty income ratio, education, body mass index, cardiovascular disease (hypertension, hyperlipemia, heartfailure, coronary heart disease, angina, and heart attack), smoking, alcohol, diabetes, and serum indicators (CRP, albumin, total cholesterol, and creatinine). Hp, Helicobacter pylori; CI, confidence interval; OR, odds ratios; Ref, reference.

Using subgroup analyses, the effects of various risk factors on the relationship between niacin intake and seropositivity against H.p were investigated. Gender, age, diabetes, hypertension, and hyperlipemia were considered for stratification. An analysis of subgroups and interaction is shown in **Fig 3**, which was in line with the findings of multivariate logistic regression.

## 4. Discussion

Our study reveals that higher niacin intake significantly reduces H. pylori positivity, especially in the upper quartiles (Q3, Q4) compared to the lowest (Q1). Even after accounting for confounding factors, this relationship remains significant. Interestingly, we identified an "L" shaped association between niacin intake and H. pylori antibodies, with a turning point at 44.69 mg/day. Below this threshold, H. pylori seroprevalence decreases with increased niacin intake, but no notable association exists at or above this level.

This discovery offers a new perspective for the non-pharmacological prevention of H. pylori infection. Although antibiotic treatment is the current standard method for managing H. pylori infection, treatment failures, recurrences, and increasing antibiotic resistance necessitate the search for alternative preventive and management strategies. Against this backdrop, our research suggests that increasing dietary niacin intake may serve as an effective preventive measure. Specifically, dietary adjustments to increase the intake of niacin-rich foods, such as meats, fish, whole grains, and legumes, can help reduce the risk of H. pylori infection. Furthermore, the promotion and implementation of this strategy could have a positive impact on public health policies. By enhancing public awareness of the role of diet and nutrition in preventing H. pylori infection, healthy eating habits can be promoted, thereby reducing the incidence of infection on a broader scale. Additionally, targeted dietary interventions for high-risk groups, such as individuals with a family history of H.pylori-related diseases, could further reduce infection risks among these populations.

This investigation represents the first exploration of the link between dietary niacin consumption and H. pylori seropositivity using a cross-sectional approach. While few studies directly address this relationship, pertinent research contributes valuable contextual understanding. Population-based retrospective cohort analysis and meta-analyses indicate that individuals with periodontitis exhibit higher rates of gastric H. pylori infection compared to those without this condition [15, 16]. Furthermore, existing research consistently supports a strong link between reduced niacin intake and the development of periodontitis, often associated with compromised immune function, elevating susceptibility to bacterial infections in the oral cavity [13, 14, 39]. Given niacin’s role in maintaining a healthy immune system, augmenting dietary niacin intake might enhance the body’s immune response and potentially reduce the likelihood of H. pylori colonization [40]. This suggests a plausible association between niacin and susceptibility to H. pylori.

The exact biological mechanisms delineating the inverse correlation between dietary niacin intake and seropositivity for H.pylori remain partly veiled. However, the corpus of existing evidence substantiates the biological plausibility of our discoveries. Mitochondrial toxicity possibly correlates with the underlying mechanism of H. pylori infection. Research precedent elucidates that the VacA toxin, secreted by H. pylori, significantly enhances the bacterium’s colonization efficiency in the stomach [41–43]. Investigation conducted by Eiki Yamasaki [44] reveals that VacA precipitates a decline in the mitochondrial transmembrane potential, thereby triggering mitochondrial toxicity. Moreover, the mitochondrial dysfunction and energy deficiency induced by VacA facilitate a more effective colonization of H. pylori on gastric epithelial cells [17, 45]. These findings suggest that mitochondrial dysfunction and energy shortage, as a result of VacA-induced mitochondrial toxicity, are pivotal in the infection process of H. pylori. It is noteworthy that increasing the intake of NAD might help alleviate conditions such as reduced mitochondrial membrane potential, decreased ATP synthesis, and energy deficiency caused by VacA [46]. Given that niacin is primarily present in the diet in the form of NAD [47], therefore, increasing dietary niacin intake to boost NAD levels could potentially counteract the mitochondrial toxicity induced by the VacA toxin, thereby reducing the risk of H. pylori infection.

Moreover, a potential correlation exists between H. pylori infection and oxidative stress. Previous studies have demonstrated that H. pylori strains can form biofilms on the surface of the gastric mucosa [48, 49]. Once formed, the biofilm can provide protection for H. pylori, helping it to survive in the gastric environment for a long time, shielded from disinfection and sterilization [18]. Bacteria’s stress response and subsequent biofilm formation can be facilitated by low concentrations of reactive oxygen species (ROS) in vivo [50]. Niacin acts as a precursor to nicotinamide adenine dinucleotide (NAD) and nicotinamide adenine dinucleotide phosphate (NADP), crucial in cellular functioning and oxidative stress regulation [10]. Studies have demonstrated that elevated niacin concentrations effectively inhibit reactive oxygen species (ROS) formation [51], and suppress superoxide production by directly reducing electron transfer [52]. Given niacin’s observed impact on cellular oxidative stress, it’s plausible to hypothesize a potential link between niacin consumption and the occurrence or progression of H. pylori infection. Niacin’s effect on cellular oxidative stress may specifically inhibit H. pylori survival or proliferation by suppressing ROS production, impeding Hp strain biofilm formation, and their adaptation to the gastric environment.

The immune modulation potentially associated with H. pylori infection involves niacin’s activation of GPR109A receptors in monocytes and macrophages, promoting CD8(+) T-cell proliferation [53]. This suggests a supplementary role for niacin in modulating immune cell functionality. Additionally, GPR109A activation in immune cells significantly influences inflammatory responses [54]. Niacin has demonstrated efficacy in reducing pro-inflammatory cytokine expression (e.g., TNFα and IL-6) in human monocytes activated by LPS and mouse macrophages [53, 55]. IL-6, secreted by macrophages in response to pathogen-associated molecular patterns (PAMPs), interacts with pattern recognition receptors (PRRs), particularly Toll-like receptors (TLRs), which hold importance in H. pylori infection [19, 56]. Hence, niacin’s modulation of immune cell function might aid in preventing H. pylori infection.

While our hypotheses stem from plausible mechanisms, further empirical studies specifically exploring the niacin-H. pylori association are needed. Investigating niacin’s impact on H. pylori colonization, survival, and the gastric environment is crucial for validating these hypotheses.

Our study’s strengths lie in its substantial cohort of 4000 participants from NHANES, a nationally representative database known for meticulous surveys and stringent quality control, ensuring high-quality data. It pioneers research on niacin intake’s link to Helicobacter pylori seropositivity, highlighting the novelty and significance of our work. Diverse methodological approaches, including dose-response curves and smoothing function analysis, fortified our findings, revealing an intriguing L-shaped relationship between dietary niacin intake and H. pylori seropositivity.

Although our research has some unique advantages, we must acknowledge that it also has certain limitations. First, this is an observational study, and despite our efforts to adjust for various confounding factors, we cannot entirely exclude the possibility of residual confounding due to unmeasured variables. Hence, our study design cannot establish causality. Second, there may be recall bias or inaccuracies in self-reporting in the two 24-hour dietary recall interviews. Moreover, our study was unable to directly measure biomarkers such as mitochondrial toxicity, limiting our ability to deeply understand the mechanisms of niacin’s action. Looking ahead, We will conduct prospective cohorts or a more complicated experimental design in future research to directly measure biomarkers related to mitochondrial function, in order to validate the effects of niacin on H.pylori infection and its potential mechanisms. Lastly, this study focused solely on the American population, which limits the generality of the results to other populations. Therefore, future research needs to be conducted in a more diverse population to verify whether our findings are universally applicable.

## 5. Conclusion

Our findings highlight a significant inverse relationship between dietary niacin intake and H. pylori seropositivity, supported by plausible mechanisms involving immunomodulatory function, mitochondrial dysfunction, and cellular oxidative stress regulation. While our study enriches comprehension of this connection, additional direct investigations are crucial to confirm and elaborate on these initial findings, potentially presenting innovative approaches for managing H. pylori infections.

## Supporting information

S1 Table Univariate logistic regression to assess the association of niacin intake with Helicobacter pylori seropositivity. Abbreviations: %, weighted proportion; Hp:Helicobacter pylori,CRP; C-reactive protein. Cardiovascular disease (heartfailure, coronary heart disease, angina, heart attack, stroke). CI: confidence interval;OR:odds ratios,Ref: reference.
(DOCX)

S1 Raw data (CSV)
