# Supplementary material for: Association between dietary niacin intake and Helicobacter pylori seropositivity in US adults: A cross-sectional study
Source: PLoS One. 2024 Aug 15;19(8):e0308686. doi: 10.1371/journal.pone.0308686 (PMC11326651; doi:10.1371/journal.pone.0308686)
Supplement: S1 Table — Abbreviations: %, weighted proportion; Hp:Helicobacter pylori,CRP; C-reactive protein. Cardiovascular disease (heartfailure, coronary heart disease, angina, heart attack, stroke). CI: confidence interval;OR:odds ratios,Ref: reference. (DOCX) [file pone.0308686.s001.docx]

| S1 Table. Univariate logistic regression to assess the association of niacin intake with Helicobacter pylori seropositivity | | |
| --- | --- | --- |
| Variable | OR_95CI | P value |
| Age | 1.02 (1.01~1.02) | <0.001 |
| Gender, n (%) |  |  |
| Male | 1(Ref) | |
| Female | 0.88 (0.78~1.00) | 0.046 |
| Education, n (%) |  |  |
| Below high school | 1(Ref) | |
| High school | 0.31 (0.26~0.37) | <0.001 |
| Above high school | 0.19 (0.17~0.23) | <0.001 |
| Marital Status, n (%) |  |  |
| Living alone | 1(Ref) | |
| Married or living with a partner | 1.08 (0.95~1.22) | 0.263 |
| PIR,n (%) |  |  |
| PIR＜1.3 | 1(Ref) | |
| PIR≥1.3 | 0.48 (0.41~0.55) | <0.001 |
| BMI,n (%) |  | |
| BMI＜25 | 1(Ref) | |
| BMI(25-30) | 1.28 (1.10~1.49) | 0.001 |
| BMI＞30 | 1.19 (1.02~1.39) | 0.029 |
| Smoke status, n (%) |  | |
| No | 1(Ref) | |
| Yes | 1.13 (1~1.28) | 0.056 |
| Alcohol, n (%) |  | |
| NO | 1(Ref) | |
| Yes | 0.80 (0.70~0.91) | 0.001 |
| Diabetes, n (%) |  | |
| No | 1(Ref) | |
| Yes | 1.87 (1.51~2.32) | <0.001 |
| Serum indicators |  |  |
| Albumin | 0.73 (0.61~0.87) | <0.001 |
| Total cholesterol | 1.00 (1.00~1.00) | 0.13 |
| Creatinine | 1.00 (0.90~1.11) | 0.972 |
| CRP | 1.07 (1.00~1.14) | 0.051 |
| Cardiovascular disease |  | |
| Hypertension, n (%) |  | |
| No | 1(Ref) | |
| Yes | 1.35 (1.18~1.54) | <0.001 |
| Hyperlipemia, n (%) |  | |
| No | 1(Ref) | |
| Yes | 1.30 (0.99~1.29) | 0.067 |
| Heartfailure, n (%) |  | |
| No | 1(Ref) | |
| Yes | 1.95 (1.34~2.83) | <0.001 |
| Coronary disease，n (%) |  | |
| No | 1(Ref) | |
| Yes | 1.31 (0.95~1.80) | 0.096 |
| Angina,n (%) |  | |
| No | 1(Ref) | |
| Yes | 1.34 (0.96~1.86) | 0.083 |
| Heart attack,n (%) |  | |
| No | 1(Ref) | |
| Yes | 1.47 (1.08~1.99) | 0.014 |
| Dietary intake |  |  |
| Niacin | 0.98 (0.98~0.99) | <0.001 |
| Abbreviations: %, weighted proportion; | | |
| Hp：Helicobacter pylori，CRP：C-reactive protein | | |
| Cardiovascular disease(hypertension, hyperlipemia, heartfailure, coronary heart disease, angina, heart attack, stroke) | | |
| CI:confidence interval；OR：odds ratios，Ref:reference | | |
